# Variances in Smoking Expectancies Predict Moment-to-Moment Smoking Behaviors in Everyday Life

**DOI:** 10.1007/s12529-024-10276-4

**Published:** 2024-04-03

**Authors:** Deanna M. Halliday, Matthew J. Zawadzki, Anna V. Song

**Affiliations:** 1 Center for Tobacco Control Research and Education, University of California, San Francisco, San Francisco, USA; 2 Department of Psychological Sciences, University of California, Merced, Merced USA; 3 Nicotine and Cannabis Policy Center, University of California, Merced, Merced USA

**Keywords:** Smoking, Tobacco, Ecological momentary assessment, Risk perceptions, Cigarettes

## Abstract

**Background:**

Many policy decisions about tobacco control are predicated on rational choice models, which posit (1) that smokers are aware of the risks of cigarettes and (2) that perceived risks have a consistent influence on continued smoking behavior. However, research shows that beliefs about smoking may be vulnerable to changes in internal and external contexts.

**Methods:**

Using ecological momentary assessment, we tested this by measuring how smokers’ (*N* = 52) beliefs about smoking varied over time. Four times per day over 1 week, participants responded to measures of smoking intentions, risk perceptions, mood and social outcome expectancies, and internal and external contextual factors.

**Results:**

We analyzed this data using multilevel modeling, finding that both smoking intentions, risk perceptions, and expectancies differed between participants as well as between moments.

**Conclusion:**

Risk perceptions and mood expectancies were a significant predictor of intentions to smoke in the next 30 min, illustrating the importance of these beliefs in decisional processes. This study was preregistered at the Open Science Foundation: https://osf.io/wmv3s/?view_only=71ad66d3ce3845fcb3bf2b9860d820c9. Our analytic plan was not preregistered.

## Introduction

Most people are aware of the potential for dire health consequences related to smoking cigarettes, including those who smoke [1]. However, this awareness of the health risks is not sufficient to guarantee successful cessation. Over 80% of those who smoke cigarettes have experienced problems with quitting and most regret ever picking up their first cigarette [2]. More so, most quit attempts in any given year will fail [3]. The complex nature of nicotine addiction and intervening environmental factors can derail successful cessation attempts, but the mechanisms by which these factors disrupt cessation are not fully understood. Specifically, it is unclear what parts of and to what degree the decisional processes that underlie smoking motivations are vulnerable to moment-to-moment changes in a persons’ daily life.

Rational choice models of decision-making, which have historically informed tobacco policy at the local, state, and federal levels, generally posit that beliefs, such as the perceived benefits and risks of smoking, are central to an individual’s decision to smoke [4, 5]. These economic analyses argue that if individuals are behaving rationally and understand the health risks of smoking, then the perceived benefits of smoking must outweigh those risks. A critical weakness of these economic postulates is that a person’s perceptions of risks and benefits are assumed to be stable and are always equally important when deciding to smoke. There have been a number of critiques of the rational choice theory [6], and although some recent smoking-related policy decisions have tempered its influence [7], the impact of the rational choice theory is still evident in existing legislation [8, 9].

### Expectancies and Perceptions in the Context of Rational Choice

People smoke because they have expectations for how cigarettes will affect them [10, 11]. These expectancies are both positive (benefits) and negative (risks) [12]. The perceived benefits that come from smoking may include improving mood and lowering stress, while the perceived risks may include health harms or the possibility of social conflict [13]. Expectancies are not a reflection of what actually happens when a person smokes, but a perception of what one thinks may happen, which may or may not occur [12, 14]. Moment-to-moment changes in some expectancies, such as the expectancy that smoking improves mood or can help with coping with negative affect, are related to lapses in smoking cessation [10, 15]. For example, in periods of greater negative affect, the expectation that smoking can improve mood is also greater, as is the urge to smoke [15].

Expectancy variance is often studied using ecological momentary assessment (EMA). Ecological momentary assessment (EMA) obtains brief, self-report assessments several times a day through the use of a smartphone application [14], thus allowing the measurement of momentary states that can be aggregated at the day and within-person levels. High levels of validity are achieved by assessing factors close in time to their occurrence thus reducing the influence of recall biases that are prone to arise with retrospective reports [14]. The researchers who have applied EMA in smoking research exemplify why the method is so well suited for this subject area. Such studies have determined that smoking behavior is influenced by negative affect [14, 16] and restlessness [17], as well as environmental factors such as the presence of other smokers, food consumption, and certain activities [17]. However, it is still not fully understood how these features of the environment or mood state contribute to the cognitive processes that immediately precede the decision to smoke.

Although it is increasingly clear that expectancies vary in relation to moment-to-moment changes, general risk perceptions about smoking are more likely to be seen as stable. Models like the Health Action Process Approach define outcome expectancies as a person’s beliefs about the outcome of engaging in a specific behavior while risk perceptions are a person’s general belief about their likelihood of or susceptibility to negative consequences of engaging in a behavior [18]. For example, the belief that smoking harms your health would be a risk perception, while the expectation that you will feel ill after smoking a cigarette would be an outcome expectancy. Overwhelmingly, research has examined risk perceptions as stable factors. In the same studies where researchers track varying expectancies, beliefs about smoking risks are rarely measured moment-to-moment [19]. In fact, risk perceptions are often only collected at baseline [20]. Researchers who study tobacco use often find that while increased risk perception leads to increased desire to quit smoking [21], further links to successful quit attempts are tenuous [21, 22]. The disconnect between smoking-related perceptions and smoking behavior is commonly interpreted in three ways. First, some interpret the disconnect as evidence of the shortcomings of theories [23, 24], such as exclusion of important factors and variables that might bridge the gap between perceptions and behavior (context, willingness, etc.). Second, others interpret the disconnect between perceptions and behavior as evidence of flawed decision-making or lack of information [25]. Finally, others interpret the disconnect not as flawed decision-making, but as an example of logical decision-making where perceptions of smoking-related benefits outweigh the perceived risks [26].

The third interpretation that smokers have determined the benefits outweigh risks holds important policy implications. Per federal regulations, health policies must undergo cost–benefit analyses conducted by economists [27]. In these analyses, economists make the argument that unrecognized benefits smokers experience outweigh the well-known health risks. As a result, the benefits rising from health policies must be discounted to account for consumer surplus, which is the lost “benefit” that consumers must be experiencing, but that the policy would deny them. In the context of smoking, this consumer surplus argument holds that smokers are well informed and to impose restrictive policies on smoking overlooks the “logical” decisions these smokers make [26]. However, the near universal regret smokers experience [2] is clear evidence that a simple cost–benefit analysis fails to explain continued smoking behavior. EMA studies looking at smoking expectancies have significantly contributed to our understanding of the role of these expectancies in moment-to-moment decisions making; however, general risk perceptions that play such an important role in health behavior models and policy are still often assumed to be stable.

#### The Current Study

We aimed to measure variations of beliefs about smoking using EMA. Participants responded to risk perception and outcome expectancy questions, reported both past and intended smoking behaviors, and provided information about internal and external context (e.g., craving for cigarettes, stress, presence of another smoker, and time since last cigarette) several times a day for a week. The smoking-related beliefs reflected common risk and benefits reported by smokers [12]. Prior to our primary analyses, we used a multilevel exploratory factor analysis that grouped our belief items into three domains: health risk perceptions, mood expectancies, and social expectancies. Our primary analyses addressed three questions: (1) how do beliefs about smoking and intentions to smoke vary, (2) which contextual factors influence smoking-related beliefs and intentions, and (3) which beliefs have the greatest impact on intention to smoke? We first determined what proportion of variance in smoking intentions (i.e., intent to smoke in the next 30 min) and beliefs can be attributed to between-person differences (e.g., demographic differences and differing degrees of nicotine dependence between participants) and within-person differences (e.g., moment-to-moment changes in contextual factors). We then used a multilevel logistic model to determine how risk perceptions and expectancies predicted intentions to smoke while controlling for between- and within-person factors.

## Methods

### Participants

We recruited a total of 52 current adult smokers from Merced County between the ages of 21 and 50 using a variety of recruitment methods including ads on Craigslist and Facebook, and physical fliers in local smoke shops. Participants were eligible if they were comfortable speaking English and smoked at least four separate days during the week. The majority of the participants smoked daily (*n* = 41; 78.85%). On days that they smoked, most participants consumed 10 or fewer cigarettes per day (*n* = 33; 63.46%). Fewer participants smoked between 11 and 20 cigarettes (*n* = 16, 30.77%), between 21 and 30 cigarettes per day (*n* = 2; 3.85%), or more than 31 cigarettes per day (*n* = 1; 1.92%). Participants were 34 years old on average (*M* = 33.99, SD = 9.03). More than half of our participants were female (65.88%), non-Hispanic white (52.93%), and employed (65.40%). Fewer than half of our participants had a college degree (32.26%) or reported a household income above $25,000 per year (48.76%). Only 11.86% of participants currently had a partner who also smoked. We also asked participants to complete the Fagestrom Test of Nicotine Dependence and most of our participants met the criteria for very low (46.52%) or low (23.82%) dependence [28].

### Procedure

Our study consisted of two parts: an intake session and the EMA data collection phase. During the intake session, we first collected the basic demographic information described above as well as measures of constructs well known to be associated with smoking status. These included questions about the smoking status of the participant’s romantic partner as well as the Fagestrom Test of Nicotine Dependence [28]. These demographic and smoking-related measures comprise the between-person differences in our later analyses.

Following the intake measures, which usually took about 30 min to complete, we gave participants explicit instructions on the EMA application through which they would take the remainder of their surveys. Participants downloaded the RealLifeExp application (LifeData, LLC., Marion, IN) and completed a short practice questionnaire for training purposes. We offered to provide any participant with a device to complete the EMA if they did not have their own. The training included details of the data collection schedules over the next 7 days. They received four semi-random notifications each day that prompted them to complete moment-to-moment measures, including the expectancy items. Participants responded to these same questions four times a day for the duration of the study, with at least 150 min between each notification. Participants had 1 h to complete the survey before it became unavailable. On the eighth day, participants received a short debriefing survey. Participants received $10 for the initial intake meeting, $50 if they completed the EMA portion of the experiment over the next week, and received a $10 bonus if they completed over 85% of their EMA responses. All participants received this compensation in the form of Amazon gift cards.

Participants could receive a maximum of 28 notifications over the week-long study; however, there was some variation in the number of notifications depending on the study start time for each participant. There was also one participant who deleted the application prior to study completion, resulting in fewer notifications. In total, 1273 notifications were sent out over the course of the study. The participants completed 69.91% of these surveys for a total of 890 completed timepoints.

### EMA Measures

#### Risk Perception and Expectancy Domains

Smoking behaviors are determined by balancing both the risks and benefits of smoking, and these consequences cover multiple domains [29]*.* We selected 11 items from prior research [12] that broadly represented three categories (i.e., health, mood, and social beliefs) as well as an indicator of decisional balance. All items were reworded to measure that expectancy at the current moment (e.g., “Right now, if I were to have a cigarette, I would feel more relaxed.”). Participants responded on a 1 (*strongly disagree*) to 7 (*strongly agree*) scale. Although we selected these items to reflect specific categories, it was unclear if these categories remain the same after we adapted the measures. We therefore used a multilevel exploratory factor analysis to test different potential factors structures across all moments (within-person) and across individuals (between-person). Model fit was determined by looking at fit indices, the extent to which identified factors were orthogonal to each other, and coherence of items on factors in line with the dimensions targeted with each item. Based on these criteria, we selected a three-factor solution (risk perceptions, mood expectancies, and social expectancies) at both levels that was a strong fit to the data: *χ*^2^ (50) = 102.57, *p* < 0.001, RMSEA = 0.034, CFI = 0.960, TLI = 0.913, SRMR (within-level) = 0.024, and SRMR (between-level) = 0.058. The fit was generally consistent across both levels. Only one item did not load strongly on any factor at the within-person level and thus was not included. As such, we created averages of these sets of items at each moment resulting in three expectancy domains (see Table 1). Domain scores ranged from 1 to 7.

**Table 1:** Belief items and results for multilevel EFA

**Items**	**Within-Person Level**			**Between-Person Level**		
	1	2	3	1	2	3
1. I believe that my next cigarette will cause harm to my health	.691*	.018	− .006	.955*	.001	.106
2. I feel that cigarettes are causing harm to my health	.531*	.034	− .021	.999*	.022	.004
3. I’m worried that smoking will cause harm to my health	.530*	− .021	.029	.755*	.006	.280
4. If I were to have a cigarette, I would feel more relaxed	.030	.699*	− .004	.042	1.031*	.009
5. If I were to have a cigarette, I would feel happier after smoking	− .006	.682*	.093	− .001	.959*	-.078
6. I believe smoking is a way to relieve stress	.030	.391*	− .100	.190	.876*	.007
7. If I were to have a cigarette, I would feel unhappy after smoking	.170	− .316*	.170	.004	− .745*	1.127
8. Smoking helps me feel connected to the people in my life	.008	.245	.475*	− .176	.068	.278*
9. I feel that smoking is a source of conflict in my relationships with people	.269	− .009	.410*	.098	− .062	.499*
10. I feel like the benefits I get from smoking outweigh the risks	.127	.259	.319*	− .537	.334	.402*
11. I am not worried about the potential long-term risks of smoking	− .043	.117	.127	− .253	.114	.398

^*^The factor loading was significant, *p* < 0.05

#### Risk Perceptions

The items that combine to contribute to our risk perception score include “I feel that cigarettes are causing harm to my health”; “I believe that my next cigarette will cause harm to my health”; and “I’m worried that smoking will cause harm to my health.” A person’s final risk perception score is the average across the three items, and a higher score indicates a greater agreement that smoking may harm health.

#### Mood Expectancies

The items that comprise our mood expectancy score include “If I were to have a cigarette, I would feel happier after smoking”; “If I were to have a cigarette, I would feel unhappy after smoking”; “If I were to have a cigarette, I would feel more relaxed”; and “I believe smoking is a way to relieve stress.” We reversed coded our second item prior to combining items. A higher score indicates a stronger belief that smoking will improve mood or reduce stress.

#### Social Expectancies

Our social expectancy score comprised the following items: “Smoking helps me feel connected to the people in my life”; “I feel that smoking is a source of conflict in my relationships with people”; and “I feel like the benefits I get from smoking outweigh the risks.” The second item was again reverse coded, and, while the final item was more so meant to be a decisional balance item, it loaded onto the social factor. A higher score on the social expectancies scale indicates that the smoker is more likely to agree that smoking is beneficial or generally positive in relationships.

#### Smoking Behavior and Intentions

Participants answered two items about past smoking behavior. First they indicated if they had smoked a cigarette today answering yes or no. When a participant answered yes, they then responded to how long it has been since they smoked (“How long has it been since you last smoked a cigarette?”) answering in hours and minutes. Responses were converted to minutes to indicate how many minutes since the last cigarette. A small number of responses (*n* = 12, 2.13%) seemed to indicate values for the prior day—defined as more than 20 h. These values were rescaled to indicate the maximum value of 14 h within the same day (i.e., scores more than 20 h were rescaled as 14 h). Dates and times were used to confirm that participants were referring to behaviors in the same day. Participants also reported on anticipated smoking behavior by responding yes (1) or no (0) to the following prompt: “Do you expect you will smoke in the next 30 min?”.

#### Context of the Moment

Our survey prompted participants to report several contextual details about the moments in which they took their surveys. Participants used scales to rate the strength of their craving to have a cigarette (1, no craving at all; 7, extremely strong craving), whether they were in the presence of another person who smoked (0, no; 1, yes), and how stressed they currently felt (1, no stress at all; 7, extremely stressed).

### Analytic Plan

In line with prior research [30], we used multilevel modeling to determine (1) how expectancies and intention to smoke vary, (2) what between- or within-person factors predict changes in expectancies and intention, and (3) how variable risk perceptions and expectancy domains relate to intention to smoke while accounting for between- and within-person differences? The smoking behavior of interest was whether the individual believed they would smoke in the next 30 min. For our first aim, we used the xtmelogit in Stata IC 15.1 to specify a three-level null model, essentially a multilevel model with no predictors, for our intentions to smoke in the next 30 min, a binary variable. We then used the mixed function to test the risk perception and expectancies independently. In these models, moments were nested within days, which were nested within participants. These null models allow us to assess intraclass correlation coefficients and show what proportion of variance in intention and expectancies are accounted for by between-person, within-person between-day, and within-person within-day differences.

For our second aim, we assessed which between- and within-person predictors were related to intentions to smoke and expectancy domains in four separate models. We included a number of predictors to the null model to determine which between-person (e.g., demographics) or within-person variables were related to intentions to smoke and our expectancy domains. Continuous predictor variables are all mean centered. Between-person variables (e.g., age) are grand mean centered and within-person variables (e.g., craving at the moment) are person mean centered. The model assessed the effects of the predictor variables on behavior and expectancies with random intercepts by participant ID and study day. For each of the variables in the models, we used a likelihood ratio test to determine whether allowing for random slopes for that variable significantly improved our model. When random slopes are included in the model, we specified the use of an unstructured covariance matrix at the between-person level so that we accounted for possible covariance in random effects. Allowing for random slopes and an unstructured covariance is standard for these types of analyses [31].

For our final aim, we used a multilevel logistic model to determine how the predictors and the expectancies relate to intentions to smoke in the next 30 min. We further included time since last cigarette in the final model to control for its effects on smoking intentions. We followed similar model-building procedures as the prior analyses.

## Results

### Variance in Intentions and Expectancy Domains

Using the null three-level logistic model, we found that over half (55.77%) of the variance of intention to smoke was accounted for by the moment-level, followed by 41.82% of the variance at the person-level, with very little at the day level (see Table 2). This indicates that smoking intention is highly dependent on within-person differences, and secondarily dependent on between-person differences. We used a null three-level linear model to run similar analyses with risk perceptions, mood, and social expectancies as our outcomes of interest. Unlike intentions, risk perceptions and expectancies were more dependent on between-person than within-person differences (see Table 2). There were high levels of variance at the person-level for all expectancies, indicating that the health, mood, and social expectancies include stable beliefs as found in prior research. Yet, anywhere from about 13 to 32% of the variance was at the within-person level, mostly at the moment level rather than day level, also indicating that these perceptions and expectancies naturally vary and thus are likely responsive to one’s internal states and environmental cues.

**Table 2:** Partitioning of variance of intentions and expectancies

	**Descriptive statistics**		**Variance partition**			**Model significance**
	*% or mean*	*SD*	Person level	Day level	Moment level	*p*-value
*Smoking Intention*						
Smoke in next 30 min	44.74% (Yes)	-	41.82%	2.41%	55.77%	*p* < *0.001*
*Beliefs*						
Risk perceptions	4.11	1.41	86.22%	1.95%	11.83%	*p* < *0.001*
Social expectancies	3.03	1.08	77.89%	2.18%	19.93%	*p* < *0.001*
Mood expectancies	4.41	1.07	68.18%	3.81%	28.01%	*p* < *0.001*

Variance partition percentages are calculated using intraclass correlation coefficients generated by the command *estat icc* in Stata IC 15.1. Domain scores ranged from 1 to 7

### Between- and Within-Person Predictors of Intentions and Expectancy Domains

Both between-person and within-person variables were significantly related to intent to smoke in the next 30 min (see Table 3). This three-level logistic model included gender, race, age, income, education, partners’ smoking status, and nicotine dependence score as between-person predictors and craving, stress, and presence of another smoker in the moment as within-person predictors. The random effect of craving in the moment significantly improved the model and was therefore included in the final analysis. Non-white smokers were less likely to intend to smoke in the next 30 min (*p* < 0.001), as were those with a partner who smoked (*p* = 0.007). Stress in the moment also marginally predicted reduced likelihood that the person would smoke (*p* = 0.049). However, both craving at the moment (*p* < 0.001) and the presence of another smoker (*p* = 0.001) increased the likelihood participants reported they would smoke in the next 30 min.

**Table 3:** Predictors of intentions and expectancies

	Smoke in next 30 min $b\left(SE\right)$	Risk perceptions $b\left(SE\right)$	Mood expectancies $b\left(SE\right)$	Social expectancies $b\left(SE\right)$
*Between person*				
Gender: male	0.28 (0.42)	− 0.41 (0.38)	0.05 (0.27)	0.02 (0.27)
Race: non-White	− 1.99 (0.52)**	− 0.87 (0.45)	− 0.31 (0.32)	0.13 (0.31)
Age^a^	0.05 (0.03	− 0.01 (0.03)	− 0.001 (0.02)	− 0.03 (0.02)
Income^a^	− 0.24 (0.14)	0.09 (0.13)	− 0.28 (0.09)*	− 0.20 (0.09)*
Education^a^	− 0.01 (0.20)	0.26 (0.19)	− 0.01 (0.14)	0.12 (0.14)
Partner smokes: Yes	− 1.64 (0.63)*	− 0.45 (0.57)	0.23 (0.40)	− 0.24 (0.41)
Nicotine dependence^a^	0.26 (0.16)	0.20 (0.19)	0.02 (0.11)	0.26 (0.11)*
*Within person*				
Craving^b^	0.68 (0.12)**	-0.01 (0.02)	0.19 (0.03)**	0.06 (0.02)**
Stress^b^	− 0.16 (0.08)*	0.002 (0.02)	0.04 (0.02)	− 0.01 (0.02)
With other smoker: Yes	0.92 (0.30)*	− 0.14 (0.07)*	0.14 (0.07)	0.0002 (0.07)
*Random effects*				
Craving	0.41 (0.14)*	**-**	0.10 (0.03)**	-
Stressed	**-**	0.07 (0.02)**	%1.%2 (0.02)**	-
*Intercepts*	b (95%CI)	b (95%CI)	b (95%CI)	b (95%CI)
Fixed intercept	0.26 (− 1.16 to 1.68)	5.23 (4.03 – 6.43)	4.50 (3.65 – 5.34)	2.97 (2.14 – 3.81)
Standard deviation of intercept at participant level	0.88 (0.58 – 1.34)	1.12 (0.90 – 1.41)	0.81 (0.64 – 1.02)	0.77 (0.61 – 0.97)
Standard deviation of intercept at day level	0.50 (0.15 – 1.61)	0.23 (0.17 – 3.22)	0.28 (0.20 – 0.38)	0.19 (0.56 – 0.64)

Significance indicated by * < 0.05; ** < 0.001

^a^Items have been grand mean centered

^b^Indicates items have been person mean centered

We then assessed which between-person and within-person variables predict changes in risk perceptions, mood expectancies, and social expectancies using a three-level linear model (see Table 3). Risk perceptions were unaffected by personal characteristics. In contrast, higher income was associated with lower mood (*p* = 0.002) and social expectancies (*p* = 0.029), and greater nicotine dependence was also associated with greater social expectancies (*p* = 0.023). As for within-person differences, participants reported they believed smoking to be less harmful to their health when they were with another smoker (*p* = 0.036). At moments where cravings for cigarettes was higher, mood expectancies (*p* < 0.001) and social expectancies were also higher (*p* = 0.001).

### Risk Perceptions, Expectancies, and Intentions

The final model was similar to the previous three-level logistic model with the addition of risk perceptions and expectancies as predictors. Risk perceptions in the moment and mood expectancies, but not social expectancies, predicted of intentions to smoke in the next 30 min. The greater the perceived risk of smoking in the moment, the less likely the participant intended to smoke in the near future. Conversely, the greater the belief that smoking would improve mood, the more likely the participant was to intend to smoke. (Table 4)

**Table 4:** Expectancies predicting intentions

	$b$	*SE*	*p*-value	95% confidence interval
*Beliefs*				
Risk perceptions	− **0.50**	**0.24**	**0.038**	− **0.96**−**0.03**
Mood expectancies	**0.48**	**0.21**	**0.026**	**0.06–0.90**
Social expectancies	− 0.04	0.21	0.858	− 0.45–0.37
*Between person*				
Gender: male	0.24	0.44	0.578	− 0.62–1.11
Race: non-White	− **2.15**	**0.53**	** < 0.001**	− **3.20**−**1.11**
Age^a^	0.02	0.03	0.439	− 0.03–0.08
Income^a^	− 0.27	0.15	0.086	− 0.57–0.04
Education^a^	− 0.34	0.20	0.084	− 0.73–0.05
Partner smokes: Yes	− **2.51**	**0.66**	** < 0.001**	− **3.80**−**1.22**
Nicotine dependence^a^	0.14	0.17	0.391	− 0.18–0.47
*Within person*				
Craving^b^	**0.58**	**0.15**	** < 0.001**	**0.29–0.86**
Stress^b^	− **0.21**	**0.10**	**0.027**	− **0.41**−**0.02**
With other smoker: Yes	0.73*	**0.34**	**0.033**	**0.06–1.41**
Time since last cigarette^b^	− 0.0004	0.001	0.696	− 0.002–0.002
*Random effects*				
Craving	**0.46**	**0.17**	**-**	**0.22–0.96**
*Intercepts*				
Fixed intercept	0.82	0.74	0.266	− 0.63–2.27
Standard deviation of intercept at participant level	0.84	0.23	-	0.49–1.45
Standard deviation of intercept at day level	0.54	0.33	-	0.16–1.82

Bold values are significant

^a^Items have been grand mean centered

^b^Indicates items have been person mean centered. The random effects of stress and time since last cigarette did not significantly improve the model, so they are not included

## Discussion

This research shows that (1) risk perceptions and expectancies vary, (2) this variance is partly driven by within-person differences from moment-to-moment, and (3) risk perceptions and mood expectancies predict smoking intentions. This both directly contradicts the prevailing rational choice models that guide policy and offers alternative explanations for why “rational” decision makers sometimes act against their own professed desires.

This vulnerability to changes in internal and external context has important implications for theory and intervention efforts. Rational choice models [32] assume that people are *Homo Economicus*, a decision-maker with perfect knowledge, perfect foresight, full capabilities to make decisional calculations, and stable preferences. The assumed stability of expectancies is a central assumption to normative, rational choice models of decision-making, which inform many of the decisions the Food and Drug Administration make concerning cigarettes, such as whether to place graphic warning labels on cigarette packages [8, 9]. However, this study joins the breadth of psychological research demonstrating that humans do not have these resources or capabilities. Smoking is not the rational choice, but the irrational choice [5]. This evidence of unstable risk perceptions could help explain the irrational choice to continue smoking cigarettes, particularly since these perceptions are vulnerable to moment-to-moment changes.

This is not the first time context has been implicated in smoking-related decision-making, but this study contributes to the literature by showing how context impacts both expectancies and general health-related risk perceptions. Compared to social and mood expectancies, natural variations in risk perceptions are investigated less often. In a meta-analysis of 61 smoking-related EMA studies, fewer than 10 assessed how beliefs about the consequences of smoking related to lapse in smoking cessation [19]. This current study found that not only did risk perceptions vary, but they also significantly predicted intentions to smoke. Our work shows that the presence of another smoker may also alter a smoker’s fundamental understanding of how harmful smoking is to one’s health, possibly lowering cognitive barriers to lighting the next cigarette. In moments when other smokers are not present, perceptions of health risk are higher and cognitive barriers may be higher. While we only tested one feature of the social environment, other social contexts could have complementary or competing effects. The presence of children, significant others, friends, or even strangers may also trigger changes in risk perceptions.

One limitation of this study is that we only selected a few within-person variables to test, and this is not sufficient to characterize the complexities of changes in context from moment to moment. Other environmental variables, such as the presence of smoking-related advertisements, may also significantly impact decision processes. However, our ultimate goal is to provide evidence that both risk perceptions and expectancies vary, as evidenced by our null models, and that EMA is an appropriate methodology to test how this variance occurs. We are also limited by participants’ patience and availability, and as such, chose to make the four daily surveys as short as possible. Future work could benefit from advanced, automatic data collection which does not require direct input from participants, such as ambulatory data collected by fitness devices, location data, or new technology that collects smoking data through wearable sensors [33]. Our primary interest in this study was to specifically look at decisional processes, but future work could use wearable devices to look directly at behavioral outcomes. The participants in this sample had generally low self-reported smoking rates and nicotine addiction indicators, yet also frequently reported intentions to smoke within the next 30 min after a notification. There was some concern that participating in this study may have increased participants’ smoking frequency beyond their typical consumption rates,however, among 38 participants who completed a debriefing survey at the end of the study, 28 (73.68%) believed they smoked the same as usual during the period of participation and 7 (18.42%) believed that they actually smoked less. Alternatively, it is possible that participants either (a) under-reported their smoking frequency, (b) did not always act on their intentions to smoke within 30 min, or (c) the semi-random notifications aligned with periods of smoking. Without objective measures of smoking frequency, we cannot resolve this discrepancy in this current study.

Besides implications for theory and policy, this work has direct implications for smoking interventions. Personalized, adaptive smoking interventions show promise in providing real-time cessation support [34]. Our work complements these efforts by revealing which cognitions may be vulnerable to which factors. Smokers experiencing strong cravings, for example, may need reminders that smoking rarely improves mood, and often worsens it instead [24]. Reminders about the risks of smoking may also be particularly beneficial in areas where social smoking is common. In-the-moment interventions may benefit by addressing cognitive variability and reaffirming the smoker’s own beliefs they professed in contexts when cravings were lower or other smokers were not present.

Mobile technology allows for ecologically valid surveillance of psychological and behavioral phenomena, allowing us to show that smoking-related cognitions are vulnerable to moment-to-moment changes in context for the first time. For the majority of smokers who want to quit smoking [2], this work provides some explanation of why they may rationalize continued smoking in moments of high craving or when with other smokers. Risk perceptions are not consistent influences on behavior, and while that contradicts some traditional theories of behaviors, this knowledge also provides new avenues for research and opportunities for in-the-moment interventions that may improve smoking cessation efforts.

## Data Availability

The data analyzed in this article are not publicly available due to the sensitive nature of the topic. However, deidentified data may be available from the corresponding author on reasonable request. Methods and materials are available at the above Open Science Foundation link.

## Code Availability

Analytic code may be available from the corresponding author on reasonable request.
